# Implementing Collaborative Writing in Chinese EFL Classrooms: Voices From Tertiary Teachers

**DOI:** 10.3389/fpsyg.2021.631561

**Published:** 2021-06-24

**Authors:** Yao Zheng, Shulin Yu, Icy Lee

**Affiliations:** ^1^School of Foreign Languages and Cultures, Chongqing University, Chongqing, China; ^2^Faculty of Education, University of Macau, Taipa, China; ^3^Faculty of Education, The Chinese University of Hong Kong, Shatin, China

**Keywords:** collaborative writing, teacher perceptions, teacher knowledge, teacher practices, teaching materials

## Abstract

While collaborative writing has been increasingly investigated in educational research, little is known about whether and how it is adopted as a pedagogical activity in classroom contexts. This exploratory study investigated EFL teachers' perceptions of the implementation of collaborative writing in Chinese tertiary institutions. The analysis of in-depth interviews with 31 EFL teachers from 13 institutions in the People's Republic of China and their teaching materials reveals mismatches between their perceptions and practices, as well as their perceptions and knowledge. While the teachers perceived collaborative writing as valuable and feasible, more than half of them were not using it, and their perceptions were not supported by sound teacher knowledge. Practical implications are provided for implementing collaborative writing in classroom contexts.

## Introduction

Collaborative writing is a pedagogical activity that allows students to complete a text through coordinated efforts, shared responsibility, and joint decision making (Storch, 2013). As a relatively novel activity in the language classroom where writing is traditionally regarded as a solitary, individual act, collaborative writing has strong theoretical support and is found to have great practical value for students (Ede and Lunsford, 1990; Wigglesworth and Storch, 2009; Shehadeh, 2011). On the theoretical front, it meshes well with the tenets of language acquisition and learning theories, such as cognitive and sociocognitive theories. The activity is also supported by communicative language teaching and task-based language teaching, which emphasize the interactions of students for language development. Practically, there is increasing evidence of the benefits of collaborative writing for student writers across different contexts, such as improving both immediate and subsequent writing performance, providing rich opportunities for language use, developing communicative competence, and facilitating long-term language learning (Shehadeh, 2011; Storch, 2013; Abe, 2019).

While there is no lack of research that discusses how collaborative tasks could be designed to teach writing, little is known about whether and how collaborative writing is adopted as a pedagogical activity in real-world contexts, especially in the second language (L2) classrooms. Previous studies have focused on students' experiences with and perspectives on collaborative writing, such as their interaction patterns, perceptions of collaborative writing (Shehadeh, 2011), and how student attitudes may impact the interaction patterns and language learning opportunities (Chen and Yu, 2019a). There is a scarcity of research on classroom teachers' perceptions and knowledge of collaborative writing, which are likely to determine whether and how teachers implement collaborative writing. Without a clear knowledge of how classroom teachers perceive collaborative writing, we could hardly obtain a nuanced understanding of how the approach can be implemented in real-world contexts.

The exploratory study presented in the article was motivated by the idea that investigating how teachers perceive collaborative writing in the language classrooms is significant because teacher perceptions reflect teacher knowledge and have a direct influence on their decision making and instructional behaviors (Chartrand and Bargh, 1999). The study set out to explore English writing teachers' perceptions of collaborative writing in Chinese tertiary contexts. A qualitative design was adopted to investigate 31 English-as-a-foreign-language (EFL) teachers from 13 institutions in the People's Republic of China (PRC). Data were gathered from interviews with individual teachers and their teaching materials. Three research questions were used to guide the present study: (1) How did Chinese tertiary writing teachers perceive collaborative writing? (2) To what extent were teachers' perceptions based on a sound knowledge of collaborative writing? (3) To what extent were teachers' perceptions reflected in their practice?

## Literature Review

### Conceptualizing Teachers' Perceptions and Knowledge of Collaborative Writing

In educational research, the concept of teacher perception has been used interchangeably with other notions, such as teacher attitudes (Hargan, 1995; Hajian et al., 2014) and teacher perspectives (Huang, 2010; Allen and Paesani, 2020). Informed by Gibson (1986) notion, teacher perception in our article refers to teachers' detection of pedagogy-related information arising from the interactions of teachers with the teaching environment. Such pedagogy-related information includes the values, benefits, and feasibility of the pedagogical approach concerned (Charalambous et al., 2014; Huizenga et al., 2017; Zhao, 2018). Following this conceptualization, in this study teachers' perceptions of collaborative writing entail their detection of information about collaborative writing through their interactions with the work milieu, reflecting teachers' views about the usefulness, benefits, and feasibility of collaborative writing.

Teacher knowledge, broadly referred to as what a teacher knows about teaching, constitutes a crucial component of teacher cognition (Borg, 2003; Yigitoglu and Belcher, 2014). Shulman (1986, 1987) has proposed a categorization of teacher knowledge that includes content knowledge, general pedagogical knowledge, curriculum knowledge, pedagogical knowledge, knowledge of learners and their characteristics, knowledge of educational contexts, and knowledge of educational ends, purposes, and values, as well as their philosophical and historical grounds. In a similar vein, Calderhead (1991) argues that teacher knowledge takes different forms and knowledge growth occurs in various areas through teachers' interactions with the environment. For language teaching, Freeman (2002) cautions that while teacher knowledge reflects teachers' mental lives and hence represents a “hidden side” of teaching, context is pivotal to understanding teacher experience and teacher knowledge. Bearing in mind that teacher knowledge takes various forms, and the present study strives to understand classroom teachers who instruct students in EFL curriculums, we view the meaning of teacher knowledge of collaborative writing as threefold. First, it refers to the extent to which a teacher knows about collaborative writing as a pedagogical approach with regard to its rationales, principles, implementation, and so on—i.e., pedagogical content knowledge, according to Shulman (1986, 1987). Second, it indicates the extent to which the teacher knows about EFL students' characteristics in collaborative writing—i.e., knowledge of learners. Third, it reveals the extent to which the teacher knows the relevance of collaborative writing to the nature of EFL courses and programs—i.e., curriculum knowledge.

### Exploring Teachers' Use of Collaborative Writing in EFL Classrooms

While the past two decades have witnessed an increased research interest in students' experiences with collaborative writing, there has not been a parallel uptake of collaborative writing activities in EFL learning settings (Storch, 2013). In light of the promise collaborative writing holds for language learning (Storch, 2005, 2019; Li et al., 2012; Li, 2013), previous research in EFL contexts has centered around student-related topics, such as their attitudes toward (Kim, 2008; Elola and Oskoz, 2010; Chen and Yu, 2019b) and interactions during collaborative writing (Li and Kim, 2016; Li and Zhu, 2017; Zhang, 2019). However, teachers' practices in utilizing collaborative student writing are under researched in the extant literature.

Despite a lack of research that scrutinizes teachers' use of collaborative writing in EFL classrooms, Storch (2013) has offered practitioners some practical guidelines about the planning and implementation of collaborative writing in real-world contexts with regard to issues about task type, group size, work allocation, and assessment. In deciding the task type for collaborative writing activities, Storch (2013) suggests that teachers should take cognizance of their own pedagogical goals and students' language proficiency, and can choose between meaning-focused tasks (e.g., jigsaw and data commentary text) and language-focused tasks (e.g., dictogloss and editing). In addition to the task type, group size and work allocation are essential to the implementation of collaborative writing. Teachers can assign students to pairs or groups depending on the class size and the mode of writing (i.e., online or face-to-face; synchronous or asynchronous) (Storch, 2013). As for assessment, teachers can integrate self and peer assessment (Frykedal and Chiriac, 2011), assigning individual grades along with group grades (Nepal, 2012; Williams, 2017), and using a mixture of individual and collaborative writing assignments (Race, 2001).

Informed by the above literature on teacher perception, teacher knowledge, and collaborative writing practice, this study seeks to investigate teachers' perceptions of collaborative writing, the extent to which their perceptions are supported by a sound knowledge of collaborative writing, and to what extent teacher perceptions are manifested in their practice.

## The Study

### Participants

Thirty-one teachers were recruited from 13 tertiary institutions using a purposive sampling approach first through the first author-researcher's personal contacts and then by snowballing methods (Yin, 2011). The teacher participants were instructors of either an English Writing course that focused exclusively on the teaching of English writing^1^ or a College English course with which English writing was integrated.^2^ The 13 institutions included six large-size comprehensive universities, two normal universities specializing in pre-service teacher training, two universities of science and technology, one medical university, one university of economics and business, and one vocational college specializing in architecture. The participants had varied teaching experiences. There were novice teachers with a teaching experience of less than 5 years and experienced teachers who had taught for more than 10 years. They were teaching students from college to postgraduate levels in both English-major and non-English-major programs. Most of the participants were Chinese, aging between 27 and 56. Two participants were international teachers who had a master's degree in TESOL (a Korean and an Indian; both non-native speakers of English). Twenty-two teachers were female and nine were male. Table 1 shows the teachers' demographic information.

**Table 1:** Interviewees' demographic information.

**Teachers**	**Institutions**	**Teaching experiences**	**Course**	**Target students**
14	6: comprehensive universities	9: ≥ 10 years 3: < 5 years 2: 5–10 years	9: College English 5: English Writing	9: FYNEU 4: SYNEU 1: FYNEG
6	2: normal universities	3: ≥ 10 years 2: < 5 years 1: 5–10 years	3: College English 3: English Writing	3: FYEU 1: FYNEU 1: SYNEU 1: FYNEG
4	2: universities of science and technology	3: < 5 years 1: ≥ 10 years	3: College English 1: English Writing	2: SYNEU 1: FYNEU 1: FYNEG
3	1: university of economics and business	2: 5–10 years 1: ≥ 10 years	2: College English 1: English Writing	2: SYNEU 1: FYNEU
2	1: medical university	1: 5–10 years 1: ≥ 10 years	1: College English 1: English Writing	1: FYNEU 1: SYNEU
2	1: vocational college	1: 5–10 years 1: ≥ 10 years	1: College English 1: English Writing	1: FYNEC 1: FYEC
In total (31 interviewees from 13 institutions)		16: ≥ 10 years 8: < 5 years 7: 5–10 years	19: College English 12: English Writing	14: FYNEU 8: SYNEU 4: FYEU 3: FYNEG 1: FYNEC 1: FYEC

*FYNEU, first-year non-English major undergraduates; SYNEU, second year non-English major undergraduates; FYEU, first-year English major undergraduates; FYNEG, first-year non-English major graduates; FYNEC, first-year non-English major college students; FYEC, first year English major college students*.

### Data Collection and Analysis

In-depth interviews were used to explore participants' perceptions, understandings, and practices of collaborative writing. Three broad questions guided the semi-structured interviews: (1) what is your understanding of collaborative writing (e.g., its definition, theoretical underpinnings, target teaching contexts, and issues to be considered in using it); (2) how do you perceive collaborative writing [e.g., its merits and (in)feasibility in your teaching context]; and (3) are you using collaborative writing in your instruction or would you consider using it? Why or why not? And how?

The first question focuses on teachers' understanding of collaborative writing, which could elicit their overall knowledge of the approach. When teachers' interview responses indicated their misunderstandings of collaborative writing, we provided explanations. For example, Wang, a female teacher working at a university in Hebei province, thought “collaborative writing is peer review” that just requires the students to “correct errors on the work of each other.” In this case, we first explained to her that collaborative writing is widely viewed as a pedagogical activity where students write a text together through coordinated efforts, shared responsibility, and joint decision making. We emphasized that our research focuses on such activity and then proceeded to the next question. The second question was designed following our operational definition of teacher perception that emphasizes the dictation of pedagogy-related information. The last question focuses on teachers' practices in using collaborative writing that take place in the classroom settings and can cast a great impact on teachers' perceptions through their interactions with the teaching environment. When asking the two questions, we paid special attention to their reasons, trying to understand not only the how but also the why of their perceptions or implementation of collaborative writing. Further knowledge of individual teachers, as well as their teaching environment, was obtained in their explanation of reasons.

Two to three interviews were conducted with each teacher (face-to-face or online via WeChat; around 40 minutes for each interview). They were conducted in Mandarin and English with the native Chinese teachers and international teachers, respectively. The first interview aimed at obtaining their personal information and general responses to the above questions and the follow-up interviews elicited further explanations on the responses. For example, Lin, a female teacher working at a university in Chongqing, reported in the first interview that she was using collaborative writing in her course. She also provided general comments on its merits such as “raising students' group awareness” and “saving teacher's effort in marking individual writing.” After the interview, we asked the teacher for sample student texts, which were used as prompts in the second interview for her to describe her practice in detail. In the third interview, we further checked her understanding of collaborative writing by asking her to provide more explanations on her practice. All interview sessions with the teachers were audio-recorded and then manually transcribed for analysis. After the interviews, six teachers voluntarily provided their sample teaching materials (e.g., PowerPoint slides and class handouts) as supporting evidence.

NVivo (v. 12) was used for the thematic analysis of interview data (Yin, 2011). Sample teaching materials were utilized when we sought contextual evidence or supplementary data. The analysis involved six stages. The first stage was the familiarization of data by reading through interview transcripts to obtain a general sense. In the second stage, open coding was performed to examine teachers' interview responses and then inductively identified three major categories, namely *understanding of collaborative writing* (i.e., knowledge), *perceptions of collaborative writing*, and *(dis)use of collaborative writing* (i.e., practice). In the third stage, axial coding was conducted by creating nodes under each category. Teacher understanding was analyzed into *defining collaborative writing, designing collaborative writing activity*, and *assessing collaborative writing*. For teacher perceptions, we built the nodes of *perceptions of the value* and *perceptions of the feasibility*. Regarding the (dis)use of collaborative writing, four nodes were created, namely *using and intending to continue using, using but hesitating to continue using, disusing but intending to use*, and *disusing and intending to continue disusing*. The creation of the nodes permitted us to obtain a systematic and nuanced understanding of the teachers' responses that led to several unifying ideas constituting themes. The fourth stage aimed to seek recurring themes through a scrutiny of the above codes first within- and cross-category and then in relation to each participant's interview transcripts. Short descriptions of the initial themes were produced for further analysis. Cells were also added for inserting snapshots of some participants' teaching materials, where relevant. The fifth stage was the identification of the most prominent themes from the initial ones. The initial themes were explored again in all the transcripts to combine similar ones and revise theme descriptions. A refined theme list was created. In the last stage, a final theme list was generated after a discussion of the refined list between the first and second researcher, and member checking with two participants (Yin, 2011). Peer debriefing was finally used to safeguard potential biases in data analysis. The themes together with preliminary findings were shared with scholars from similar research backgrounds at a conference on English language teaching in PRC. Relevant feedback was used for subsequent revisions in interpreting and presenting the main findings.

## Findings

With a view to exploring tertiary EFL teachers' perceptions of collaborative writing, the findings of our study are presented in three prominent themes: (1) teachers perceived collaborative writing as valuable and feasible, (2) teacher knowledge did not match teacher perceptions, and (3) teacher practices did not match teacher perceptions. The themes are reported in the following section, with examples taken from the participants' interview data and teaching materials. Quotes of Chinese teachers' interview responses were translated into English, and those of international teachers were transcribed verbatim.

### Teachers Perceived Collaborative Writing as Valuable and Feasible

Teacher perceptions of collaborative writing have mainly to do with teachers' views of the value and benefits of collaborative writing and the extent to which collaborative writing is appropriate and feasible in their teaching context. Our findings reveal that all the teachers perceived collaborative writing as useful in changing the climate in the writing classroom because it could help them shift their traditional role as classroom dominator to another one that is a facilitator. With the shift, the students would take greater responsibility for learning. Six teachers also believed that collaborative writing provides an impetus for them to engage in reflective practice, making them realize that students can have a lot of unique contributions to make to the writing process as they write collaboratively. For example, Jing, teaching in a national key university, commented on students' contributions in the following quote.

#### Interview Response 1

Collaborative writing provides students with opportunities to give and receive peer instructions through interactions. They can help each other in solving problems, improving proficiency, and filling knowledge gaps. They could do what the teacher could not do, such as supporting each other after class. (Jing)

Moreover, 28 teachers considered that collaborative writing is an appropriate activity for tertiary students, who need to be equipped with collaborative skills to cope with the demands of the workplace. Also, as students progress from high school to tertiary education, they are expected to demonstrate greater competence in their learning and academic performance. As such, collaborative writing comes in useful, according to the participating teachers, and it is in line with the general aims of tertiary education. Interview Response 2 is from Kim, a Korean teacher who had obtained his Master's degree in TESOL in the U.S. and then taught in a large-scale Chinese university for 3 years. It gives a glimpse of the teachers' perception of collaborative writing as a highly appropriate activity for tertiary students.

#### Interview Response 2

My students have already been trained to be mature English learners in high schools. […] They come to the university to be better learners, to learn more abilities. It [collaborative writing] is appropriate! (Kim)

Regarding teacher perceptions of the feasibility of using collaborative writing, 24 out of 31 teachers agreed it could be implemented for English instruction in Chinese tertiary institutions, especially in computer-aided learning environments. Cloud-based academic writing tools (e.g., iWrite), online synchronization service (e.g., OneDrive and QQ document), and automated writing evaluation programs (e.g., Pigai and Grammarly) were mentioned when they commented on the feasibility. Tong, teaching in a university of science and technology, explained his perceptions in the following response.

#### Interview Response 3

From the space to the classroom, countless things have become possible in the digital world, not to mention a teaching approach. […] Students nowadays are all *Di Tou Zu* [低头族, people who are easy to be distracted by their mobile phone or similar device]. But I think it's OK. There are numerous Apps [applications] available on their smartphones that they can use for learning activities. They are genuine “Internet natives.” (Tong)

While technology was viewed as a powerful, facilitative tool for collaborative writing, teacher scaffolding was mentioned as a prerequisite for successful implementation. Nan, a teacher instructing non-English major graduates, stated how he perceived the students should be guided to understand writing genres and then work with each other in writing.

#### Interview Response 4

The first step should be modeling. The teacher may want to first demonstrate how he/she analyzes a specific genre and then decide the collaborating process. Many things need to be considered in the process, such as who should obtain what kind of information to share and write. (Nan)

To summarize, all teachers thought that collaborative writing is a valuable pedagogical activity, and the majority of them perceived it as feasible to implement the activity in their contexts. They further perceived that technology and teacher scaffolding play a crucial role in facilitating its implementation.

### Teacher Knowledge Did Not Match Teacher Perceptions

Surprisingly, although teacher scaffolding was perceived as essential in implementing collaborative writing and the teachers also perceived themselves as able to provide it, their knowledge of the approach was found to be limited. Eleven out of the 31 teachers could not provide proper interpretations as to what collaborative writing constituted. Some perceived it broadly as students working together for group composition and some construed it narrowly as brainstorming or peer editing (e.g., Wang). The findings suggest that the nature and distinctive features of collaborative writing were not well-recognized.

**Figure 1:**
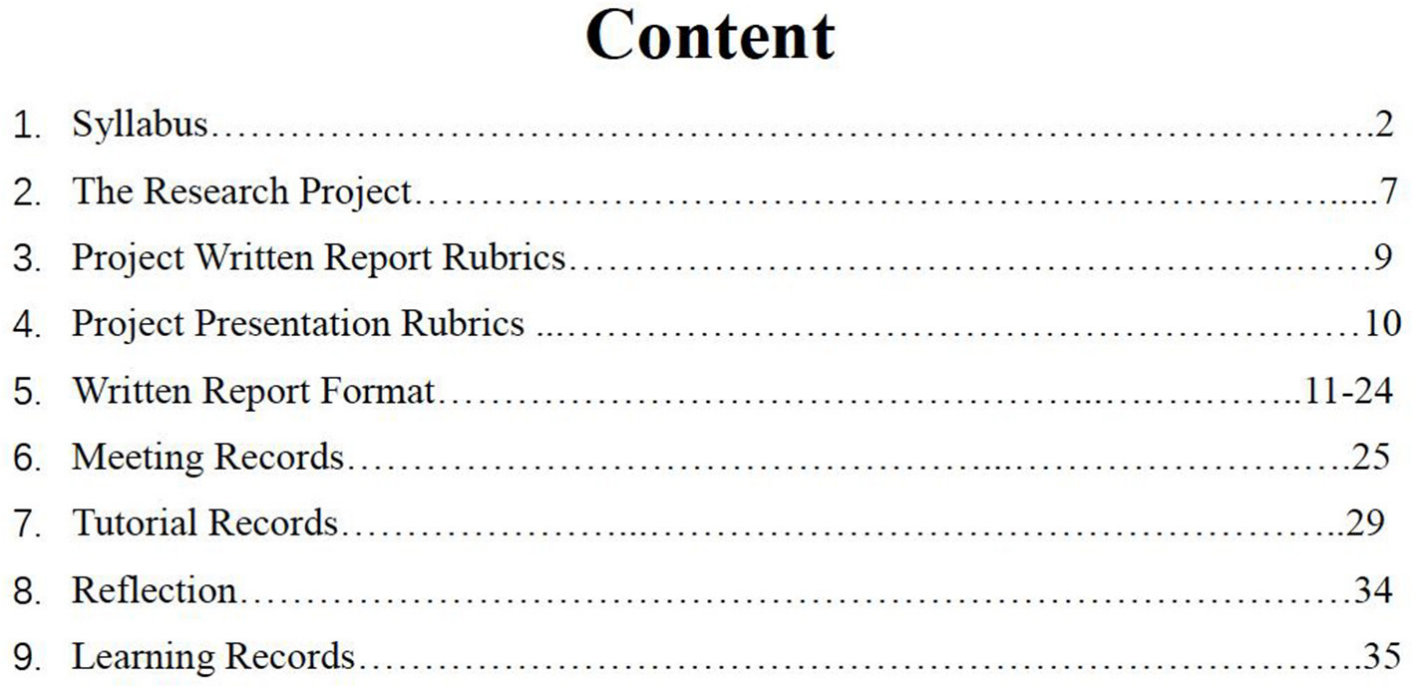
Snapshot of the Content of Lin's Student Handbook.

Figure 1 provides a snapshot of the content of a teacher's writing handbook for her students. Lin taught the College English course at a research-oriented national key university, during which she guided students to conduct authentic research in groups to explore topics that they found interesting and relevant to their daily lives (e.g., environmental protecion). To help students complete their research essays, Lin employed collaborative writing activities after class. Despite her effort in providing form-focused scaffolding to the students, as indicated in the detailed descriptions of relevant rubrics and formats in the handbook (see Figure 1), little support was provided to the students in terms of how to work together throughout the composing process. Lin further admitted in the interviews that she did not give any instructions or training on how students could finish the project report collaboratively. The quotes below are illustrative.

#### Interview Response 5

I give them the handbook to read after class because in the class I don't have enough time for those things [collaborative writing activities]. I may comment a little bit in the class, for example, on their writing formats or linguistic errors. (Lin)

#### Interview Response 6

To be frank, I have never thought about providing additional training to students on the activities. I just give them a writing assignment and they finish it collectively or split the work. You see, almost everything they need to know is listed in the handbook, such as the formats and rubrics. (Lin)

Further analysis of the participating teachers' understanding of collaborative writing revealed that their limited knowledge might be attributed to their limited exposure to the pedagogical approach. According to their responses, none of them had received any formal training on using collaborative writing. Coupled with the lack of training was the teachers' own limited collaborative writing experience. Only three teachers mentioned having written collaboratively with others. They all had a Ph.D. degree, and their prior collaborative writing experience was working with other scholars for academic publications. However, it was not clear how they actually understood and approached collaborative writing back then. Lee, who had a Ph.D. degree in Education and only 1 year of teaching experience, further admitted:

#### Interview Response 7

It's good, but…Even though I have prior experience of writing collaboratively for academic publications, I don't know how to teach my students to do so. Neither writing nor collaborative learning is my expertise. I focus on listening and speaking research […] Literally, I don't even know how to teach writing without a coursebook. I was assigned to teach the course without any training on writing pedagogies. (Lee)

Overall, although collaborative writing was perceived as practicable and teacher scaffolding as crucial to its implementation, the teachers lacked knowledge of the approach in terms of its essence, key features, and main procedures. Their perceptions of collaborative writing were not based on a sound understanding of its theoretical rationales, grouping strategies, and assessment of the process and product.

### Teacher Practices Did Not Match Teacher Perceptions

Interestingly, although all the teachers were favorably inclined toward collaborative writing, not all of them were using it and/or intended to adopt it in their classroom. Table 2 presents the teachers' use/disuse of collaborative writing practice. Results indicate that more than half of them were not using the approach (16; 52%), while a large proportion of teachers were neither using nor intended to use it in the future (13; 42%). Although several teachers were using collaborative writing and intended to continue with it (10; 32%), some currently using it admitted that they were hesitant about adopting the activity (5; 16%). Only a small portion of those without experience with collaborative writing stated that they wanted to try it in future teaching (3; 10%).

**Table 2:** Summary of collaborative writing practice.

**Collaborative writing practice**		**No**.	**Percentage**
Disusing		16	52%
	Disusing and intending to continue disusing	13	42%
	Disusing but intending to use	3	10%
Using		15	48%
	Using and intending to continue using	10	32%
	Using but hesitating to continue using	5	16%

It is interesting to note that the majority of teachers had no intention to adopt collaborative writing or were using it with hesitation, even though all of them perceived it as useful for writing instruction. Focusing on the interview responses of those not using or intending to discontinue the approach, we found that the main obstacle was contextual constraints. For example, Yang, a novice teacher at a province-level normal university who had only taught for 3 years, was considering dropping collaborative writing for the multiple pressure she was facing.

#### Interview Response 8

I'm facing great pressure from both the faculty and my students. In my first 2 years of teaching, our faculty send some more experienced teachers to observe my classes. They offered me suggestions from an “expert perspective.” I was told by one of them that I should not spend much time on student group work. Instead, more teacher instructions were needed in the class, as she did. Otherwise, the faculty would view me as lazy and not fulfilling my responsibilities. […] Now I feel my students are not motivated to conduct the activity. They think their primary purpose in attending the class is to listen to the teacher. Literally speaking, listen to the teacher; just as how they did in high schools. It's the teacher's job to talk and instruct all the time. They would not be happy if the task is assigned to them but I just monitor because they are not used to such kind of classroom activities. (Yang)

The above interview response shows that Yang was caught in a conflict between the social expectations of the dominant role of an EFL teacher and learner-centred pedagogy epitomised by collaborative writing. Simply put, while her faculty and students expected a teacher-driven culture of learning that gives prominence to knowledge transformation from the teacher to students, collaborative writing put her students in charge of their learning. Given the pressure, Yang was not motivated to continue using the approach.

Yang's response was corroborated by that of Dong, a more experienced teacher who taught at the same university but did not use collaborative writing. Although Dong perceived that collaborative writing is valuable and could be feasible at some universities, he was discouraged by the contextual constraints within his own institution.

#### Interview Response 9

I know it's theoretically feasible, but we don't have a favorable environment to support teachers' use of collaborative writing here. [..] For example, we have thousands of students to teach every semester. There are usually more than 10 teachers instructing the course [College English], and each one is in charge of six to eight classes. As required by our faculty, the teachers should follow a course syllabus provided by the English department. The syllabus specifies the coursebook to be used, themes and topics to be covered, activities to be conducted, and, most importantly, the assessment. Only individual work is assessed according to the syllabus, and the final term exam concentrates on the coursebook's contents. […] If there is one teacher who would like to use collaborative writing in teaching, the teacher must be very careful in allocating the time for the activities because there is much to cover in the coursebook. I believe it would result in much more trouble than just focusing on the coursebook.

Dong decided not to use collaborative writing because of the “trouble” of using an activity that could not fit well into their course syllabus. “I believe there are other teachers who share the same thought,” he said.

## Discussion

While previous research has emphasized students' experiences in collaborative writing, teachers' voices are also vital in understanding the effective implementation of the approach. As shown in the findings of this study, although the teachers perceived that collaborative writing was valuable and feasible, they tended not to use the approach or used it with hesitation. Moreover, their perception was not supported by a sound teacher knowledge of collaborative writing. This probably explains why collaborative writing remains a vibrant activity in research but is not embraced by the majority of L2 teachers in real classrooms. The mismatch between teachers' perception and knowledge of collaborative writing adds new knowledge to the current literature dominated by studies about students' performance in (e.g., Shehadeh, 2011; Chen and Yu, 2019a) and teachers' practice of collaborative writing (e.g., Wette, 2015; Alghasab et al., 2019). Indeed, without scrutinizing and understanding teachers' perceptions and knowledge, it is hard for insights from collaborative writing research to trickle down to the classroom to influence teachers' practice. This exploratory study is the first step toward understanding teachers' perceptions, understanding, and knowledge of collaborative writing.

The findings of our study reveal that though the teachers perceived collaborative writing as useful, appropriate, and feasible, their perceptions were reflected to a limited extent in their practices. While previous research has found students' language proficiency to be a big barrier to collaborative writing (e.g., Zhang, 2018), the present study shows that it was the sociocultural context that discouraged some teachers from undertaking collaborative writing. For example, Yang, a novice teacher who had tried collaborative writing, was uncertain if she should continue with the practice. When implementing collaborative writing in her class, she faced pressure from different sources, such as the faculty administrators who seemed to lack a proper way to evaluate teacher endeavors, her colleagues who viewed collaborative writing as against the institutional policies, and her students who were accustomed to the traditional teacher-dominated classroom (see Interview Response 8). Yang was not alone in struggling with the pressure because four other teachers who were using collaborative writing reported similar obstacles. Two teachers who had never used the activity in their classes admitted that the main reason for not using it related to their work milieus that did not favor the implementation of collaborative writing. They also felt that teachers in their contexts were not fully entrusted to take control of their pedagogical practice and there were always institutional policies imposed on them. The dilemma of those EFL teachers could be meaningful in better interpreting Storch's (2011) statement that the use of collaborative writing activities in L2 classes seems relatively limited. While teacher reluctance to use the approach may be one of the reasons for the limited use of collaborative writing in L2 classrooms (Storch, 2013), contextual constraints could be another one that hinders the wide implementation of collaborative writing.

Another barrier to the use of collaborative writing relates to teachers' limited knowledge of the approach. It should be noted that while our study follows Shulman's (1986, 1987) categorization of teacher knowledge that includes seven knowledge forms, teacher knowledge of collaborative writing is a synthesis of the forms that reflects the extent to which a teacher knows about collaborative writing as a pedagogical approach, EFL students' characteristics when they write collaboratively, and the relevance of the approach to the nature of EFL courses and programs. In the present study, however, around one-third of the teacher participants (*N* = 11) in the interviews did not have proper interpretations of collaborative writing. The activity was perceived by some teachers as equal to any tasks that involve students working together regardless of whether a written text is produced or not. Even for teachers who used collaborative writing, few of them demonstrated a clear understanding of the principles in determining the task type, group size, work allocation, and assessment. This suggests a lack of teacher pedagogical content knowledge of collaborative writing. While the teachers perceived teacher scaffolding as essential in implementing collaborative writing, they did not explain how the scaffolding should take place to address the characteristics of their EFL students in collaborative writing. “I just give them a writing assignment and they finish it collectively or split the work,” as Lin said. It seems that the teachers did not know what went on when their students wrote collaboratively and were unsure about students' needs in the activities. Furthermore, the teachers were unable to see how collaborative writing could be integrated seamlessly into the writing curriculum to enhance learning and teaching. For those who used the approach, no sophisticated grasp of teaching materials that facilitate the implementation of collaborative writing and cater to the curricular needs was found.

In light of the major findings, two practical implications could be drawn from this study. First, the development of collaborative writing in real-world contexts should be based on sound teacher knowledge of collaborative writing. Such knowledge includes pedagogical content knowledge of the approach, such as its benefits, principles, and procedures of implementation; knowledge of learners, which foregrounds teachers' understanding of the characteristics of EFL students in collaborative writing; and knowledge of curriculum, which enables teachers to see how collaborative writing could be integrated seamlessly into EFL courses. To equip teachers with such knowledge, training should be provided. Teacher training may include an emphasis on the rationales of the approach, its merits and potential challenges, grouping strategies, and interaction patterns. During the training, teachers could be shown classroom examples of collaborative interactions and encouraged to write collaboratively with others. The exposure and hands-on experience are conducive to a better teacher understanding of the activity. The research literature on students' experiences in collaborative writing, theories of curriculum development and collaborative writing, and sample cases of course designs are also valuable resources that could be utilized in the training.

The second practical implication concerns the need to establish a favorable environment for teachers to experience success with collaborative writing. Although the teachers in this study perceived collaborative writing as valuable and feasible, they were discouraged by the negative attitudes of students and colleagues. Research has found that a sustainable environment for pedagogical innovations often requires the support of key stakeholders such as students and principal members of the larger institution (e.g., Shehadeh, 2011; Storch, 2013; Chen and Yu, 2019a). Changing mindsets is easier said than done, but teachers with a strong conviction of collaborative writing could get together and undertake classroom-based research to produce findings in support of their practice. With collaborative efforts, they can present a strong case to the university management to let them understand the purpose, nature, process, and benefits of collaborative writing.

## Conclusion

Aiming to contribute evidence of EFL teachers' perceptions and use of collaborative writing in classroom contexts, the present study draws on in-depth interviews with teachers at Chinese tertiary institutions and their teaching materials. The findings suggest that while all the teachers perceived collaborative writing as valuable in their classrooms, not all of them adopted it, revealing a misalignment between their perceptions and practice. Although most of them perceived collaborative writing as feasible, more than half of them were not using it, and their perceptions were not supported by a sound teacher knowledge of the approach.

Given the exploratory nature of the study, as well as its limitations, the findings have to be interpreted with caution. First, our participants included novice and experienced teachers, Chinese and international teachers, but we did not investigate if these differences could influence their perceptions of collaborative writing. Future research may scrutinize whether prior experience or language background may influence teachers' perceptions of collaborative writing. Second, the present study revealed some potential to collaborative writing but did not examine how teachers could get around the obstacles. To shed light on how collaborative writing can be implemented in real-world contexts, more classroom-based research should be conducted and show how teachers learn to implement the innovative approach and grapple with constraints in their situated contexts.

## Data Availability Statement

The raw data supporting the conclusions of this article will be made available by the authors, without undue reservation.

## Ethics Statement

The studies involving human participants were reviewed and approved by Research Service and Knowledge Transfer Office at University of Macau (Reference NO. SSHRE19-APP054-FED). The patients/participants provided their written informed consent to participate in this study.

## Author Contributions

Data of the study were collected by YZ, who also analyzed the initial data. SY and IL worked with her in further data analysis and drafting the manuscript. All authors contributed to the article and approved the submitted version.

## Conflict of Interest

The authors declare that the research was conducted in the absence of any commercial or financial relationships that could be construed as a potential conflict of interest.

## Footnotes

^1^The courses were provided to undergraduate or postgraduate students for English writing development. They were referred to as Creative English Writing, Academic English Writing, or Basic/Advanced English Writing in some institutions.

^2^In 2007, the Ministry of Education in PRC issued a document that mandated College English for first- and second-year undergraduates majoring in subject areas other than English.
